# MeCP2 deficiency results in robust Rett-like behavioural and motor deficits in male and female rats

**DOI:** 10.1093/hmg/ddw179

**Published:** 2016-06-21

**Authors:** Kelsey C. Patterson, Virginia E. Hawkins, Kara M. Arps, Daniel K. Mulkey, Michelle L. Olsen

**Affiliations:** 1 Department of Cell, Developmental, and Integrative Biology, University of Alabama at Birmingham, Birmingham, AL, USA; 2 Department of Physiology and Neurobiology, University of Connecticut, Storrs, CT, USA

## Abstract

Since the identification of *MECP2* as the causative gene in the majority of Rett Syndrome (RTT) cases, transgenic mouse models have played a critical role in our understanding of this disease. The use of additional mammalian RTT models offers the promise of further elucidating critical early mechanisms of disease as well as providing new avenues for translational studies. We have identified significant abnormalities in growth as well as motor and behavioural function in a novel zinc-finger nuclease model of RTT utilizing both male and female rats throughout development. Male rats lacking MeCP2 (*Mecp2*^ZFN/y^) were noticeably symptomatic as early as postnatal day 21, with most dying by postnatal day 55, while females lacking one copy of *Mecp2* (*Mecp2*^ZFN/+^) displayed a more protracted disease course. Brain weights of *Mecp2*^ZFN/y^ and *Mecp2*^ZFN/+ ^rats were significantly reduced by postnatal day 14 and 21, respectively. Early motor and breathing abnormalities were apparent in *Mecp2*^ZFN/y^ rats, whereas *Mecp2*^ZFN/+ ^rats displayed functional irregularities later in development. The large size of this species will provide profound advantages in the identification of early disease mechanisms and the development of appropriately timed therapeutics. The current study establishes a foundational basis for the continued utilization of this rat model in future RTT research.

## Introduction

Rett Syndrome (RTT) is an X-linked neurodevelopmental disorder affecting approximately 1 in 10 000 girls annually (1) that is caused by mutations in the gene encoding the transcriptional regulator MeCP2 in over 95% of typical cases (2–5). The syndrome is marked by seemingly normal development until 6–18 months of age, at which point language, motor, social and cognitive skills regress, cephalic growth decelerates, and hand stereotypies and breathing abnormalities appear (5–8). Although emerging longitudinal data suggest that females with RTT may live well into their fifties (9), the disease nonetheless has devastating impacts on quality of life.

Historically, RTT has been studied primarily through the use of genetically manipulated mouse models with alterations in the *Mecp2* gene (10–22), all of which display a range of abnormal neurologic phenotypes. Overall, these models have been relatively successful in recapitulating many of the features observed in the clinical progression of RTT (for an extensive review of existing RTT mouse models, see references (23–26). Although many studies thoroughly examined phenotypes in overtly “symptomatic” male and less frequently female animals, few have fully characterized these models beginning early in development. Given that RTT is a pervasive developmental disorder presenting with an initial period of “normality” followed by severe regression and subsequent pseudostationary and deterioration phases, it is important to remain cognizant of the pathophysiologic changes that may occur in any model throughout each stage of development. This is especially critical when considering the potential for a targeted therapeutic window early in disease progression.

Although mice have long been the preferred species in the modelling of genetic disorders, relatively recent advances in the ability to manipulate the rat genome offer unique opportunities, especially in the study of central nervous system (CNS) diseases like RTT. Relative to the mouse, the increased size of the rat, and subsequently all of its anatomical structures, offers obvious advantages in a surgical setting, but confers additional benefits when considering the importance of structure size and spatial relationship in both region-specific and age-dependent studies of the CNS. Electrophysiological studies and direct drug delivery experiments, for example, are considerably easier when dealing with large rat structures, an effect that is magnified if studies are performed at very early developmental stages. It is also well established that, compared to mice, rats demonstrate a wider variety of more complex cognitive and social behaviours for which testing paradigms are available and validated (27–35). All of these factors make the use of a rat model an appropriate choice for the study of a neurodevelopmental disorder such as RTT, in which deficits of higher cognitive functioning and social interaction predominate, and examination at early developmental time points may be critically relevant to therapeutic potential. Aside from these specific advantages, the addition of a rat model to the collection of resources currently available in the study of RTT will provide a platform for validating and comparing behavioural phenotypes in different species with different genetic backgrounds, thus making possible a more complete understanding of complex disease mechanisms which will facilitate translatable pharmaceutical development.

With these considerations in mind, we sought to establish the utility of a novel zinc-finger nuclease rat model of RTT, generated by Sage laboratories, by characterizing its motor and behavioural phenotypes that recapitulate the fundamental features observed in existing RTT mouse models as well as human RTT patients. This model contains a 71 base pair deletion in exon 4 of the *Mecp2* gene, resulting in the absence of MeCP2 protein. As one of the first characterizations of the motor and behavioural phenotypes in this model, we felt it important to follow both male and female rats through the entirety of their development. We herein report that brain weight is reduced in this RTT rat model by postnatal day (PND) 14, both motor and behavioural deficits are clearly observed as early as the fourth postnatal week, and these deficits continue throughout development.

## Results

### *Mecp2* transcript and protein levels are altered in *Mecp2*^ZFN/y^ and *Mecp2*^ZFN/+ ^rats

**Figure 1.**
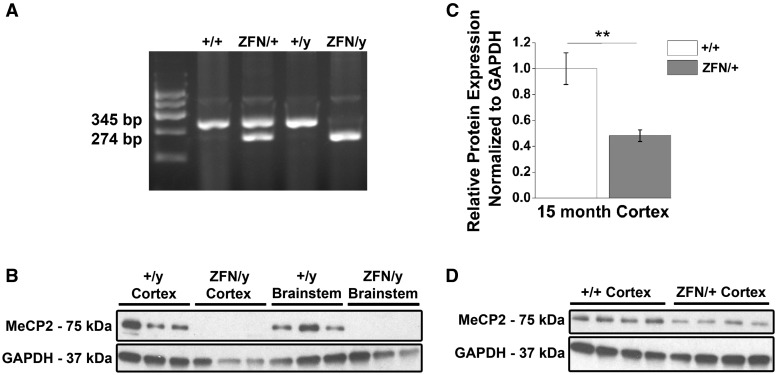
*Mecp2*^ZFN/y^ male and *Mecp2*^ZFN/+ ^female rats display altered MeCP2 protein expression. **(A)** Representative genotyping gel revealing DNA fragments of different lengths that differentiate WT from *Mecp2*^ZFN/y^ and *Mecp2*^ZFN/+^ rats. **(B)** Western blotting demonstrates total absence of MeCP2 protein in both cortex and brainstem of PND 60–80 *Mecp2*^ZFN/y^ rats. **(C–D)** 15-month-old *Mecp2*^ZFN/+^ rats show a ∼52% reduction in MeCP2 protein by western blotting of cortical tissue (WT *n = *4 *Mecp2*^ZFN/+^ *n = *4). Data are presented as mean ± SE, with asterisks representing significant genotype differences (**P < 0.01).

Sprague Dawley females lacking one copy of *Mecp2* (Sage labs) were created using zinc-finger nuclease technology that resulted in a 71 base pair deletion in the 4^th^ exon of the *Mecp2* gene. Breeding of these female rats to wildtype (WT) Sprague Dawley males produced progeny in the expected Mendelian ratios. Genotyping differentiated WT animals from male rats lacking MeCP2 (*Mecp2*^ZFN/y^) and female rats lacking one copy of *Mecp2* (*Mecp2*^ZFN/+^) (Fig. 1A). Utilizing a C-terminus targeted antibody, western blot analysis of tissue isolated from the cortex and brainstem indicated a complete absence of full length MeCP2 in *Mecp2*^ZFN/y^ rats as compared to WT males (Fig. 1B). Cortical MeCP2 protein expression was significantly reduced by 52% in 15-month-old *Mecp2*^ZFN/+ ^rats relative to WT females (Fig. 1C–D). In a complementary study by Veeratagaven *et al.* western blot analysis utilizing an N-terminus targeted antibody also confirmed the absence of truncated MeCP2.

### *Mecp2*^ZFN/y^ males and *Mecp2*^ZFN/+ ^rats display altered brain and body weights early in development

**Figure 2.**
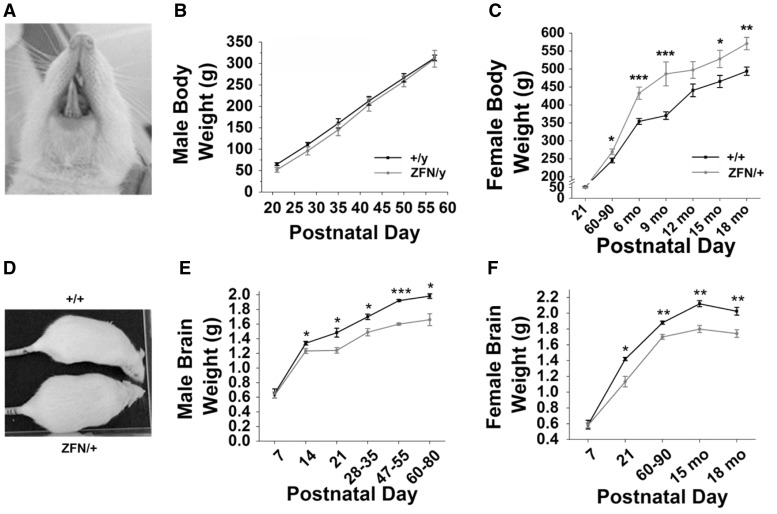
*Mecp2*^ZFN/y^ and *Mecp2*^ZFN/+ ^rats display altered body and brain weights throughout development. **(A)** Representative example of early malocclusion in a PND ∼28 *Mecp2*^ZFN/y^ rat. **(B)** *Mecp2*^ZFN/y^ body weight does not significantly differ from WT body weight throughout development. (PND 21 WT *n = *13 *Mecp2*^ZFN/y^ *n = *11; PND 28 WT *n = *12 *Mecp2*^ZFN/y^ *n = *10; PND 35 WT *n = *7 *Mecp2*^ZFN/y^ *n = *8; PND 42 WT *n = *7 *Mecp2*^ZFN/y^ *n = *8; PND 50 WT *n = *13 *Mecp2*^ZFN/y^ *n = *11; PND 57 WT *n = *11 *Mecp2*^ZFN/y^ *n = *9; PND 64 WT *n = *3 *Mecp2*^ZFN/y^ *n = *4) **(C–D)** *Mecp2*^ZFN/+^ females are significantly heavier than WTs beginning at postnatal day 60 and continuing through 18 months of age (PND 21 WT *n = *21 *Mecp2*^ZFN/+^ *n = *25; PND 60–90 WT *n = *23 *Mecp2*^ZFN/+^ *n = *22; 6 mo WT *n = *21 *Mecp2*^ZFN/+^ *n = *25; 9 mo WT *n = *14 *Mecp2*^ZFN/+^ *n = *14; 12 mo WT *n = *12 *Mecp2*^ZFN/+^ *n = *13; 15 mo WT *n = *13 *Mecp2*^ZFN/+^ *n = *13; 18 mo WT *n = *5 *Mecp2*^ZFN/+^ *n = *5). **(E)** *Mecp2*^ZFN/y^ males display significantly decreased brain weight beginning at postnatal day 14 and continuing throughout development. (PND 7 WT *n = *5 *Mecp2*^ZFN/y^ *n = *7; PND 14 WT *n = *5 *Mecp2*^ZFN/y^ *n = *6; PND 21 WT *n = *6 *Mecp2*^ZFN/y^ *n = *6; PND 28–35 WT *n = *6 *Mecp2*^ZFN/y^ *n = *9; PND 47–55 WT *n = *9 *Mecp2*^ZFN/y^ *n = *10; PND 60–80 WT *n = *6 *Mecp2*^ZFN/y^ *n = *5). **(F)** *Mecp2*^ZFN/+^ females demonstrate significantly decreased brain weight beginning at postnatal day 21 and continuing through 18 months of age. (PND 7 WT *n = *4 *Mecp2*^ZFN/+^ *n = *7; PND 21 WT *n = *5 *Mecp2*^ZFN/+^ *n = *3; PND 60–90 WT *n = *5 *Mecp2*^ZFN/+^ *n = *7; 15 mo WT *n = *5 *Mecp2*^ZFN/+^ *n = *6; 18 mo WT *n = *8 *Mecp2*^ZFN/+^ *n = *7). Data are presented as mean ± SE, with asterisks representing significant genotype differences (*P < 0.05, **P < 0.01, ***P < 0.00 1).

In evaluating the general phenotype of these animals, we sought to determine if *Mecp2*^ZFN/y^ or *Mecp2*^ZFN/+ ^rats displayed alterations in either body or brain growth, as has been previously reported in knockout and *Mecp2* heterozygous mice (10–13,16,19–22,36–44). One obstacle in the evaluation of this data proved to be the development of dental malocclusion (Fig. 2A) in approximately 80% of *Mecp2*^ZFN/y^ rats (data not shown) by PND 50. This was infrequently observed in *Mecp2*^ZFN/+ ^rats during the 18-month period of data collection, and this trait was never observed in WT animals of either sex (data not shown). For all studies described herein, animals that demonstrated active malocclusion were excluded from further testing. Body weights of *Mecp2*^ZFN/y^ rats that did not develop malocclusion by PND 50 did not differ significantly from WTs at any of the developmental time points examined (Fig. 2B). Interestingly, many *Mecp2*^ZFN/y^ rats that went on to develop malocclusion were generally smaller than their WT counterparts starting early in development, before tooth lengthening. In general, *Mecp2*^ZFN/y^ rats displayed a normal habitus but felt, upon handling, hypotonic and limp when compared to WT littermates (see Supplementary Material, video 1, where the two less active animals are *Mecp2*^ZFN/y^ and the two most active males are wildtypes). *Mecp2*^ZFN/+ ^rats displayed a tendency for weight gain, becoming significantly heavier than WTs by PND 60-90 and maintaining an endomorphic habitus, on average, throughout development (Fig. 2C and D). *Mecp2*^ZFN/+ ^rats also displayed a subjectively increased incidence of hypotonia, especially at later time points. Evaluation of wet brain weights revealed that *Mecp2*^ZFN/y^ brains weighed significantly less than their WT littermates as early as PND 14 (Fig. 2E). Similarly, the brains of *Mecp2*^ZFN/+ ^rats weighed significantly less than that of WT littermate females beginning at PND 21 (Fig. 2F).

### *Mecp2*^ZFN/y^ and *Mecp2*^ZFN/+ ^rats display diminished overall wellness and decreased survival

**Figure 3.**
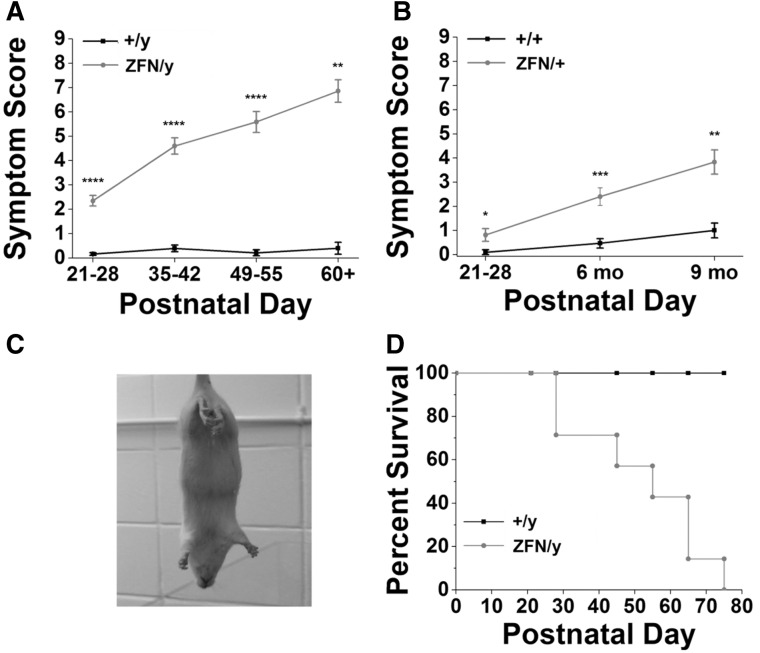
*Mecp2*^ZFN/y^ and *Mecp2*^ZFN/+ ^rats display diminished overall wellness and decreased survival. **(A)** *Mecp2*^ZFN/y^ males and **(B)** *Mecp2*^ZFN/+^ females display increased overall symptom scores relative to WTs throughout development (Males: PND 21–28 WT *n = *43 *Mecp2*^ZFN/y^ *n = *46; PND 35–42 WT *n = *23 *Mecp2*^ZFN/y^ *n = *25; PND 49–55 WT *n = *19 *Mecp2*^ZFN/y^ *n = *17; PND 60+ WT *n = *5 *Mecp2*^ZFN/y^ *n = *7; Females: PND 21–28 WT *n = *10 *Mecp2*^ZFN/+^ *n = *16; 6 mo WT *n = *15 *Mecp2*^ZFN/+^ *n = *15; 9 mo WT *n = *7 *Mecp2*^ZFN/+^ *n = *12). **(C)** Representative photograph of hindlimb clasping in an adult *Mecp2*^ZFN/y^ rat. **(D)** A Kaplan–Meier survival curve demonstrates that half of *Mecp2*^ZFN/y^ males have died by PND ∼55 (WT *n = *11 *Mecp2*^ZFN/y^ *n = *7). Data are presented as mean ± SE, with asterisks representing significant differences (*P < 0.05, **P < 0.01, ***P < 0.001, ****P < 0.0001).

**Figure 4.**
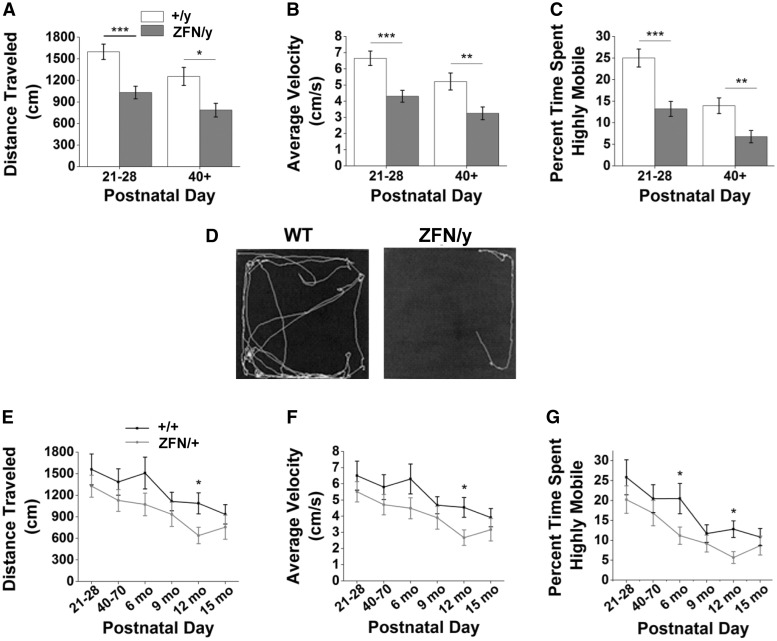
*Mecp2*^ZFN/y^ and *Mecp2*^ZFN/+ ^rats display altered locomotion upon open field testing. **(A–C)** *Mecp2*^ZFN/y^ males demonstrate a significantly decreased distance travelled (A), average velocity (B), and time spent in a highly mobile state (C) when compared to WTs at both early and late developmental time points (PND 21–28 WT *n = *31 *Mecp2*^ZFN/y^ *n = *24; PND 40+ WT *n = *21 *Mecp2*^ZFN/y^ *n = *21). **(D)** Representative tracings of open field movement for PND 40+ WT (left) and *Mecp2*^ZFN/y^ (right) males. **(E–G)** *Mecp2*^ZFN/+^ females display a significantly decreased distance travelled (E) and average velocity (F) at 12 months of age when compared to WT females, as well as (G) decreased time spent in a highly mobile state at 6 and 12 months of age (PND 21–28 WT *n = *11 *Mecp2*^ZFN/+^ *n = *12; PND 40–70 WT *n = *10 *Mecp2*^ZFN/+^ *n = *10; 6 mo WT *n = *11 *Mecp2*^ZFN/+^ *n = *15; 9 mo WT *n = *10 *Mecp2*^ZFN/+^ *n = *12; 12 mo WT *n = *10 *Mecp2*^ZFN/+^ *n = *10; 15 mo WT *n = *10 *Mecp2*^ZFN/+^ *n = *12). Data are presented as mean ± SE, with asterisks representing significant genotype differences (*P < 0.05, **P < 0.01, ***P < 0.001).

A symptom score was generated for the animals used in this study by weekly monitoring for general wellness, and the presence and severity or absence of specific characteristics. Possible symptom scores ranged from 0 to 9 (Table 1), with WTs typically scoring close to 0. We did not score the presence of tremors, head nodding, and seizures in male and female animals, although these features were observed on occasion during handling and behavioural testing (see Supplementary Material, video 2 for *Mecp2*^ZFN/y^ and Supplementary Material, video 3 for *Mecp2*^ZFN/+^). *Mecp2*^ZFN/y^ rats demonstrated symptoms relatively early in development, with many displaying a “scruffy” fur appearance and a general lethargy compared to their WT littermates. Infrequently, *Mecp2*^ZFN/y^ rats displayed malocclusion as early as PND 21. *Mecp2*^ZFN/y^ males occasionally exhibited retracted testes as early as PND 21, and the appearance of a shrivelled scrotum often continued throughout adulthood (see Supplementary Material, video 1 for examples of this trait). *Mecp2*^ZFN/y^ rats developed symptoms quickly, and deteriorated rapidly, with symptom scores averaging above 5 by 3 weeks post-weaning (Fig. 3A). *Mecp2*^ZFN/+ ^females displayed a more gradual increase in symptom score from PND 21–28 through 9 months of age, with the average score being between 3 and 4 for 9-month-old *Mecp2*^ZFN/+ ^rats (Fig. 3B). WT male and female symptom scores above 0 were typically due to a mild developmental decrease in activity level, which is reflected in Figure 4A–G, or by fur loss around the face and dorsal body, which followed a pattern of cagemate-initiated barbering that has previously been reported in rodents (45). Hindlimb clasping (Fig. 3C), which is considered an identifying characteristic of symptomatic RTT mice, was observed in approximately 70% of *Mecp2*^ZFN/y^ and *Mecp2*^ZFN/+ ^rats by PND 50 and 9 months of age, respectively (data not shown). For an example of this feature, see Supplementary Material, video 4, in which a WT male is presented first, followed by a *Mecp2*^ZFN/y^ male demonstrating hindlimb clasping and wide-based gait.

**Table 1.** Symptom score generation. Table summarizing the score generation paradigm used to calculate a weekly symptom score for every animal reported

Symptom	**Score (Total = 0–9)**		
	0	1	2
**Activity level**	Normal (rearing, cage exploration, cagemate interaction)	Lethargic (no rearing, minimal exploration and interaction)	Little to no movement (appears lifeless or asleep)
**Hindlimb clasp**	Absent	Present	–
**Breathing abnormalities**	Absent	Present	–
**Low-clearance gait/waddle**	Absent	Present	–
**Dirty/unkempt coat, Piloerection**	Absent	Present	–
**Urine staining around genitalia**	Absent	Present	–
**Porphyrin around eyes/nose**	Absent	Present	–
**Fur loss**	Absent	Present	–

In order to address findings of decreased RTT animal survival in previous studies (10–13,20,21,37), a Kaplan-Meier survival curve was constructed from only those *Mecp2*^ZFN/y^ rats that died of causes unrelated to nutritional status or littermate aggression, and compared to WT littermates. This curve demonstrates that approximately half of all *Mecp2*^ZFN/y^ males died by PND 55 (Fig. 3D). Of note is the observed incidence of *Mecp2*^ZFN/y^ on *Mecp2*^ZFN/y^ littermate aggression, which accounted for many cases of severe open flank and hindquarter wounds that resulted in abrupt death or necessitated sacrifice (data not shown). A Kaplan-Meier curve was not constructed for *Mecp2*^ZFN/+ ^rats, as none of these animals died spontaneously of disease-related causes throughout the period of study; however, several were sacrificed due to severe obesity leading to an inability to groom, and 2 (> 12 months of age) were sacrificed due to severely deteriorating body condition.

### *Mecp2*^ZFN/y^ and *Mecp2*^ZFN/+ ^rats display altered locomotion upon open field testing

Due to the general lethargy observed in *Mecp2*^ZFN/y^ rats beginning at an early age and continuing through development (see Supplementary Material, videos 1 and 4), and *Mecp2*^ZFN/+ ^females at later time points, we sought to evaluate the overall locomotor activity by use of the open field test. We began our studies using *Mecp2*^ZFN/y^ rats and compared them with WT littermates at PND 21–28 (early symptomatic) and PND 40+ (late symptomatic) time points. *Mecp2*^ZFN/y^ males displayed a decrease in overall distance travelled, average velocity, and percent of total time spent highly mobile when compared to WT littermates (Fig. 4A–C). A representative activity tracing from one trial in late symptomatic animals is shown in Figure 4D. In contrast to WT animals, which demonstrate substantial exploratory behaviour, *Mecp2*^ZFN/y^ littermates moved little, if at all, from the location of their placement in the centre of the open field for the duration of the trial. This limited locomotor pattern made the evaluation of anxiety phenotype by the measurement of time spent in the arena centre inappropriately influenced by motor deficits, and thus unsuitable for analysis. *Mecp2*^ZFN/+ ^rats displayed a trend toward a decrease in distance travelled, average velocity, and time spent highly mobile, with these parameters showing genotype-specific statistical significance at only one or two time points between the age of weaning and 15 months (Fig. 4E–G).

### *Mecp2*^ZFN/y^ and *Mecp2*^ZFN/+ ^rats display motor abnormalities throughout development

We frequently observed *Mecp2*^ZFN/y^ and *Mecp2*^ZFN/+ ^rats displaying a “waddling” gait, where the hindlimbs swung widely about the base or appeared to partially drag on the floor in their homecages or the open field arena surface during testing. We therefore next pursued two locomotor tests, rotor rod and Catwalk™, to assess motor abnormalities.

**Figure 5.**
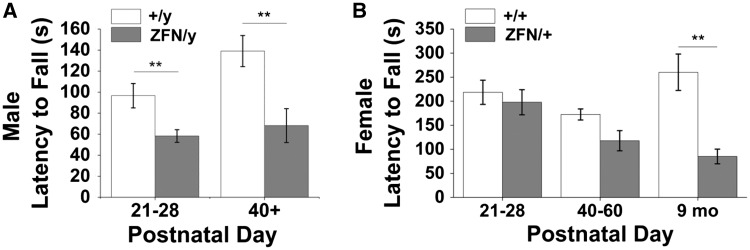
*Mecp2*^ZFN/y^ and *Mecp2*^ZFN/+ ^rats display deficits upon rotor rod testing. **(A)** *Mecp2*^ZFN/y^ males fall from the rotating rod significantly sooner than WTs at both early and late developmental time points. (PND 21–28 WT *n = *17 *Mecp2*^ZFN/y^ *n = *25; PND 40+ WT *n = *16 *Mecp2*^ZFN/y^ *n = *15) **(B)** 9 month old *Mecp2*^ZFN/+^ females display a decreased latency to fall when compared to age-matched WTs. (PND 21–28 WT *n = *8 *Mecp2*^ZFN/+^ *n = *8; PND 40–60 WT *n = *10 *Mecp2*^ZFN/+^ *n = *10; 9 mo WT = 7 *Mecp2*^ZFN/+^ *n = *6) Data are presented as mean ± SE, with asterisks representing significant genotype differences (**P < 0.01).

Unlike open field testing, which assesses the general activity level of a freely moving animal, rotor rod tests motor coordination while providing motivation by exploiting a natural fear of falling off of the rotating rod. We observed a significant decrease in latency to fall in *Mecp2*^ZFN/y^ rats relative to WT littermates at both early (PND 21–28) and late (PND 40+) developmental time points (Fig. 5A). *Mecp2*^ZFN/+ ^females also demonstrated a decreased latency to fall at 9 months of age compared to WT littermate controls (Fig. 5B). Testing of females older than 9 months of age was not assessed due to the frequency of obesity in the *Mecp2*^ZFN/+ ^population, which made placement on the rotating rod not possible.

**Figure 6.**
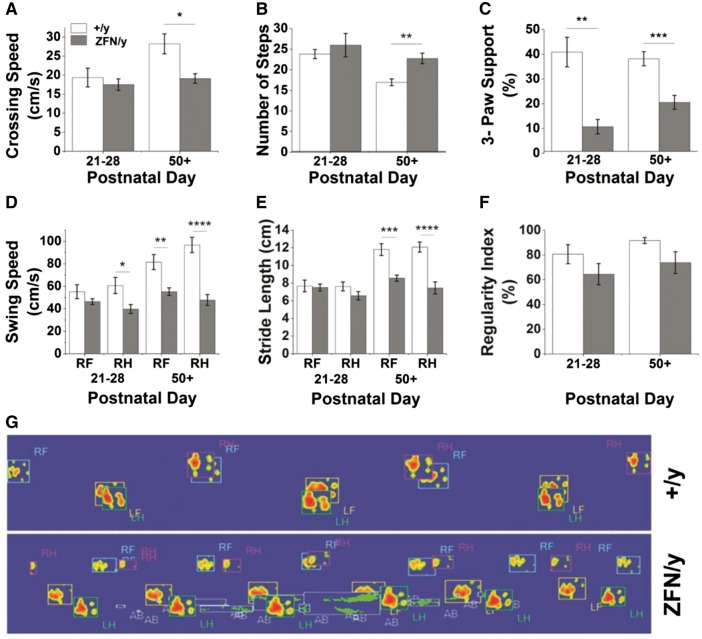
*Mecp2*^ZFN/y^ males display motor abnormalities upon Catwalk^TM^ testing as early as PND 21. **(A–B)** *Mecp2*^ZFN/y^ males cross the runway slower (A) and with a greater number of steps (B) than WTs. **(C)** *Mecp2*^ZFN/y^ males show differences in 3-paw support type at PND 21–28 and PND 50+. **(D–E)** Right hind swing speed was significantly decreased in *Mecp2*^ZFN/y^ males at PND 21–28, and right front and right hind swing speed (D), as well as right front and right hind stride length (E) were significantly decreased at PND 50+. **(F)** No significant differences were seen in regularity of step pattern between *Mecp2*^ZFN/y^ males and WTs. **(G)** Representative image of footsteps of WT (top) and *Mecp2*^ZFN/y^ male (bottom) rats as they cross the Catwalk^TM^ at PND 50+; R/L F/H = right/left front/hind, AB = abdomen. (PND 21–28 WT *n = *10 *Mecp2*^ZFN/y^ *n = *10; PND 50+ WT *n = *12 *Mecp2*^ZFN/y^ *n = *14). Data are presented as mean ± SE, with asterisks representing significant differences (*P < 0.05, **P < 0.01, ***P < 0.001, ****P < 0.0001).

Given the abnormal gait we observed in male and female animals, we additionally pursued more complex gait analysis via Catwalk™ software in order to identify potential alterations in footfall patterns and limb support among *Mecp2*^ZFN/y^ and *Mecp2*^ZFN/+ ^populations. Where applicable, right-sided motor traits were evaluated, as no lateralization of deficits was expected in this disease model. Like rotor rod, Catwalk™ provides motivation for animal movement, in this case via the placement of a dark box at one end of a narrow glass plate onto which the animal is initially placed at the opposite open-ended side. Catwalk™ testing of *Mecp2*^ZFN/y^ rats demonstrated a decreased crossing speed and an increased number of steps taken to cross the glass walkway at PND 50 and beyond relative to WT littermates (Fig. 6A–B). Although a lack of significant change in crossing speed between younger WT and *Mecp2*^ZFN/y^ males may seem contrary to previously mentioned findings of decreased velocity detected in open field testing at this age, this may be explained by the added motivational factor present in Catwalk™ testing but absent in the open field. *Mecp2*^ZFN/y^ rats displayed a decreased incidence of gait involving the simultaneous contact of 3 paws at both early (PND 21–28) and late (PND 50+) time points (Fig. 6C). Additionally, right hindlimb swing speed was decreased in *Mecp2*^ZFN/y^ rats beginning at PND 21–28, and both right front and hindlimb swing speed and stride length were decreased at PND 50 and beyond (Fig. 6D–E). Despite these observed differences between *Mecp2*^ZFN/y^ and WT males, there was no significant genotype-specific alteration in the regularity index, a measure of the normality of footfall patterns, at either time point evaluated (Fig. 6F). It is important to consider, however, that a repeating footfall pattern of any type, even if not demonstrated in the WT population, contributed to an increased regularity index in *Mecp2*^ZFN/y^ rats. Thus, *Mecp2*^ZFN/y^ males seem to maintain some type of regularity during locomotion, potentially mediated through alterations in support type, as mentioned above. The significant differences in *Mecp2*^ZFN/y^ paw placement are shown in a representative Catwalk™ tracing (Fig. 6G) as well as in Supplementary Material, videos 5 (wildtype) and 6 (*Mecp2*^ZFN/y^), where dragging of the abdomen can also be noted as an indicator of the aforementioned low-clearance gait.

**Figure 7.**
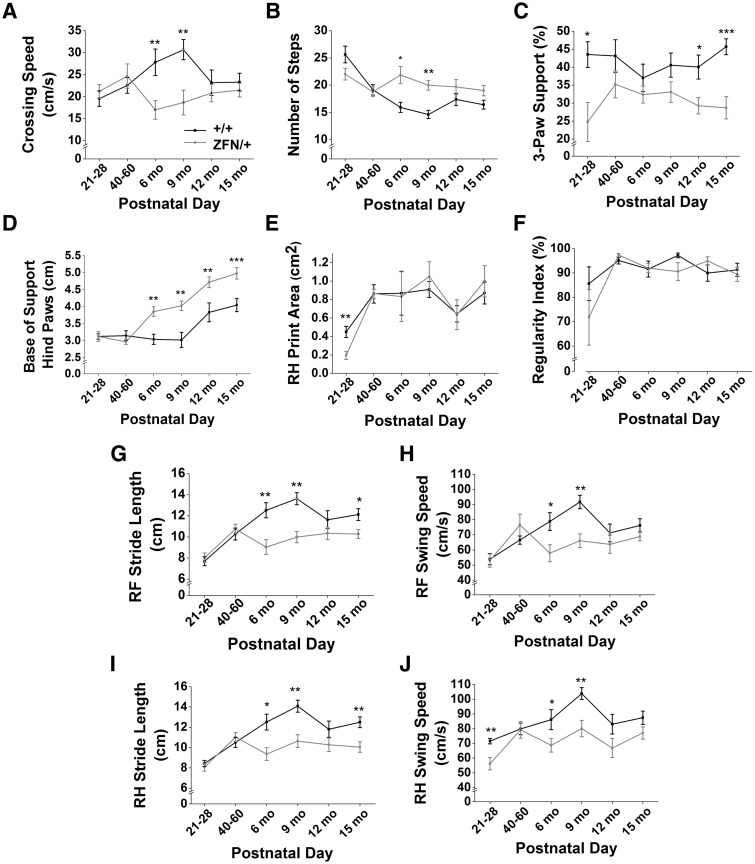
*Mecp2*^ZFN/+ ^females display motor abnormalities upon Catwalk^TM^ testing as early as PND 21. **(A–B)** *Mecp2*^ZFN/+^ females cross the runway slower (A) and with a greater number of steps (B) than WTs. **(C)** *Mecp2*^ZFN/+^ females show differences in 3-paw support type beginning at PND 21–28. **(D)** The base of support between the hind paws is significantly increased in *Mecp2*^ZFN/+^ females beginning at 6 months of age. **(E)** Right hind print area is significantly decreased in *Mecp2*^ZFN/+^ females at PND 21–28. **(F–J)** Regularity index (F) is not significantly altered in *Mecp2*^ZFN/+^ rats, although right front (G) and right hind (I) stride length and right front (H) and right hind (J) swing speed were significantly decreased in these females at various points throughout development. (PND 21–28 WT *n = *14 *Mecp2*^ZFN/+^ *n = *10; PND 40–60 WT *n = *10 *Mecp2*^ZFN/+^ *n = *10; 6 mo WT *n = *11 *Mecp2*^ZFN/+^ *n = *11; 9 mo WT *n = *11 *Mecp2*^ZFN/+^ *n = *9; 12 mo WT *n = *10 *Mecp2*^ZFN/+^ *n = *10; 15 mo WT *n = *13 *Mecp2*^ZFN/+^ *n = *13). Data are presented as mean ± SE, with asterisks representing significant differences (*P < 0.05, **P < 0.01, ***P < 0.001).

Catwalk™ testing revealed that *Mecp2*^ZFN/+ ^rats displayed significant deviations from WT motor patterns at various time points, with relatively clear trends toward alterations throughout development. *Mecp2*^ZFN/+ ^crossing speed was decreased (Fig. 7A) and number of steps increased at both 6 and 9 months of age (Fig. 7B). Usage of 3-paw support during movement was decreased in *Mecp2*^ZFN/+ ^rats at one week post-weaning as well as 12 and 15 months of age (Fig. 7C). The base of support, or width of stance, between hind paws was found to be increased in *Mecp2*^ZFN/+ ^rats beginning at 6 months of age and continuing through 15 months of age (Fig. 7D), although the higher weight of *Mecp2*^ZFN/+ ^females may have contributed to leg splay in such a manner that was not controlled for in these experiments. However, the base of support of the front paws did not differ between *Mecp2*^ZFN/+ ^and WTs throughout development, suggesting that the hindpaw stance width increase in *Mecp2*^ZFN/+^ rats may be a true compensatory gait alteration. While right hind print area was decreased in *Mecp2*^ZFN/+ ^rats only at PND 21–28 (Fig. 7E), and right front print area did not differ, true relative print areas are difficult to compare due to the significantly larger average weights of *Mecp2*^ZFN/+ ^rats beginning at PND 60–90 (Fig. 2D), which may have artificially inflated values relative to WTs. Regularity index did not differ between WTs and *Mecp2*^ZFN/+ ^rats at any point throughout development (Fig. 7F); however, forelimb stride length and swing speed were decreased in *Mecp2*^ZFN/+ ^females relative to WT at 6 and 9 months, with stride length remaining decreased at 15 months of age (Fig. 7G–H). Identical trends held true for hindlimb stride length and swing speed (Fig. 7I–J).

### *Mecp2*^ZFN/y^ rats display respiratory abnormalities early in development

**Figure 8.**
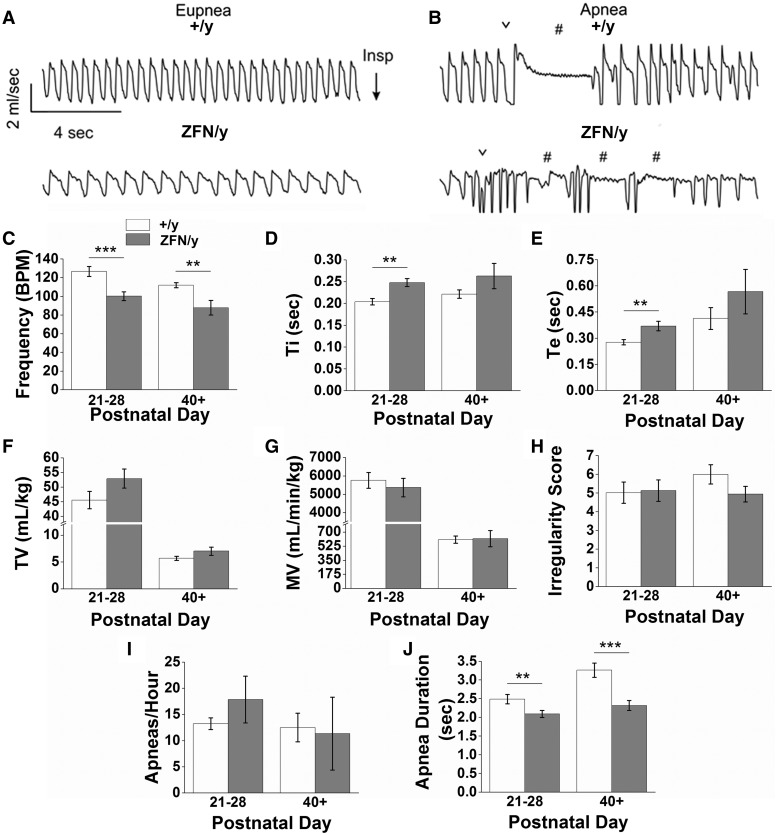
*Mecp2*^ZFN/y^ males display breathing abnormalities consistent with RTT. **(A–B)** Representative raw data plethysmography traces illustrate (A) eupnic breathing and (B) spontaneous apneic events in WT (top tracings) and *Mecp2*^ZFN/y^ (bottom tracings) juvenile males (PND 21–28). Summary data show that juvenile and adult (PND 40+) *Mecp2*^ZFN/y^ rats hypoventilate **(C)** but with no change in TV **(F)**, MV **(G)**, or respiratory irregularity **(H)** (PND 21–28 WT *n = *8, *Mecp2*^ZFN/y^ *n = *11; PND 40+ WT *n = *6, *Mecp2*^ZFN/y^ *n = *7). Consistent with reduced respiratory frequency, juvenile *Mecp2*^ZFN/y^ rats also showed an increase in Ti **(D)** and Te **(E)**. Data are represented as mean ± SEM, with asterisks representing significant differences (*P < 0.05, **P < 0.01, ***P < 0.001). ∨ designates sigh-like augmented breaths; # identifies apneic events.

Respiratory activity was measured in juvenile male (PND 21–28), adult male (PND 40+), and aged (18 month) female rats by whole-body plethysmography. We found that under control conditions (awake and breathing room air) juvenile *Mecp2*^ZFN/y^ rats exhibited abnormal breathing compared to WTs (Supplementary Material, video 7, Fig. 8A–B). Juvenile WTs breathed at a rate of 126.6 ± 5.3 BPM, which is comparable to respiratory rates of similarly aged Sprague-Dawley rats (46); however, age-matched *Mecp2*^ZFN/y^ rats breathed at a rate of only 100.1±4.7 BPM (Fig. 8A and C). The reduction in frequency was due to both an increase in inspiratory time (Ti) (0.20 ± 0.01 s WT vs. 0.25 ± 0.01 s *Mecp2*^ZFN/y^) (Fig. 8D) and expiratory time (Te) (0.28 ± 0.01 sec WT vs. 0.37 ± 0.03 s *Mecp2*^ZFN/y^) (Fig. 8E). Despite these changes in respiratory frequency, juvenile *Mecp2*^ZFN/y^ males exhibit an otherwise normal tidal volume (45.6 ± 3.0 ml/kg WT vs. 53.0 ± 3.2 ml/kg *Mecp2*^ZFN/y^) (Fig. 8F), minute ventilation (5758 ± 425.0 ml/min/kg WT vs. 5368 ± 505.4 ml/min/kg *Mecp2*^ZFN/y^) (Fig. 8G), a relatively stable respiratory cycle (irregularity score = 5.0 ± 0.6 WT vs. 5.1 ± 0.6 *Mecp2*^ZFN/y^) (Fig. 8H), and a similar number of spontaneous apneas (Fig. 8I), but of a shorter duration compared to controls (2.5 ± 0.1 s WT; 2.1 ± 0.1 s *Mecp2*^ZFN/y^) (Fig. 8J and B).

**Figure 9.**
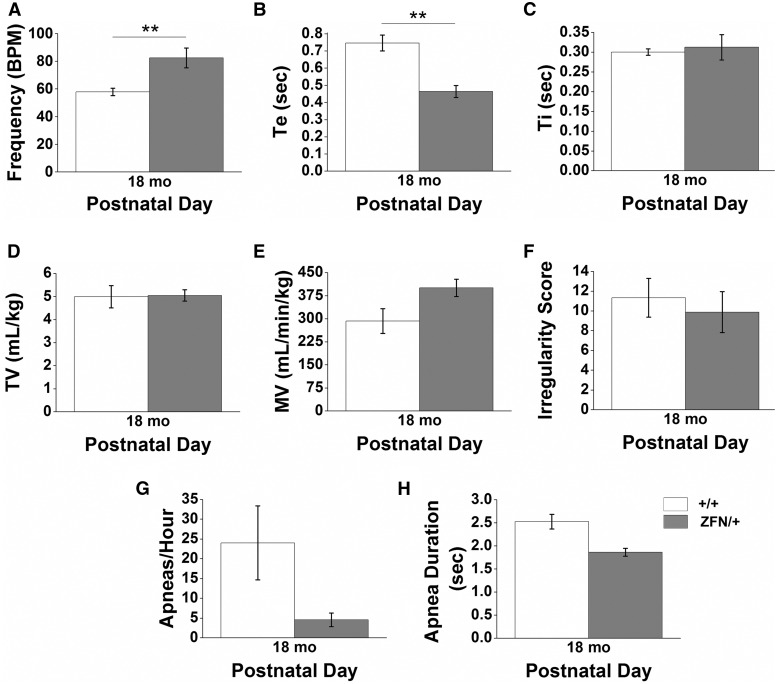
Aged *Mecp2*^ZFN/+ ^females display breathing abnormalities. Adult (18 month) *Mecp2*^ZFN/+^ females hyperventilate **(A)**, with corresponding decreases in Te **(B)** relative to WT females (PND 18 mo WT *n = *5 *Mecp2*^ZFN/+^ *n = *5). **(C–H)** TI (C), TV (D), MV (E), irregularity score (F), apnea occurrence (G), and apnea duration (H) did not significantly differ between *Mecp2*^ZFN/+^ females and WTs. Data are represented as mean ± SEM, with asterisks representing significant differences (**P < 0.01).

Respiratory activity was also measured in adult (PND40+) *Mecp2*^ZFN/y^ males and aged (18 months) *Mecp2*^ZFN/+ ^females. The respiratory phenotype of adult (PND40+) *Mecp2*^ZFN/y^ rats was similar to that described above for juvenile animals, i.e., hypoventilation (111.9 ± 2.8 BPM WT vs. 87.7 ± 7.8 BPM *Mecp2*^ZFN/y^) (Fig. 8C) and reduced apnea duration (3.3 ± 0.2 sec WT vs. 2.3 ± 0.1 *Mecp2*^ZFN/y^) (Fig. 8J), but with otherwise normal outcome measures (Fig. 8D–I). Conversely, aged *Mecp2*^ZFN/+ ^females show an enhanced respiratory frequency (57.8 ± 2.7 BPM WT vs. 82.5 ± 7.2 BPM *Mecp2*^ZFN/+^) (Fig. 9A) in conjunction with reduced expiratory time (Te) (0.75 ± 0.05 sec WT vs. 0.46 ± 0.04 sec *Mecp2*^ZFN/+^) (Fig. 9B). All other outcome measures were normal in *Mecp2*^ZFN/+ ^females (Fig. 9C–H).

Regardless of age or gender, apneic events were typically preceded by a sigh-like augmented breath in both WT and MeCP2-deficient animals (Fig. 8B); however, unlike WT animals, *Mecp2*^ZFN/y^ rats showed little expiratory component associated with this augmented breathe, perhaps suggestive of breath-hold like behaviour. It is worth noting anecdotally that juvenile and adult WT rats showed spontaneous apneic events in a stochastic manner, whereas age matched *Mecp2*^ZFN/y^ rats tended to have multiple apneas consecutively (Fig. 8B). For example, only 0% and 2.7% of apneas observed in juvenile and adult WT rats occurred within 5 sec of another apnea, whereas 15% of all apneas observed in age matched *Mecp2*^ZFN/y^ rats occurred within 5 sec of another apnea. This tendency for consecutive apneas is consistent with an unstable pattern of breathing.

## Discussion

This rat study represents the first RTT developmental and behavioural evaluation of its kind outside of traditional murine models of the disease. Our data indicate that male RTT rats demonstrate a complete absence of MeCP2 protein and an early onset of rapidly progressive disease, with ∼50% dying by PND 55. No *Mecp2*^ZFN/y^ rat in our study survived past PND 72. *Mecp2*^ZFN/+ ^rats show an approximate 50% reduction in MeCP2 protein and a more protracted, variable, and less severe disease progression than that of affected males. Our data indicate that the current rat model displays a variety of phenotypic characteristics reminiscent of classical RTT, which positions it as a valuable mammalian model for future studies. A relative comparison of this model to other murine RTT models is provided in Table 2.

**Table 2:** RTT Murine Model phenotype comparison. Table comparing the various behavioural phenotypes examined in this RTT rat model to the earliest reported abnormal findings for each of these features in various RTT mouse models to date

	**RTT rat model (current study)**		**Earliest reported RTT mouse models**	
Feature evaluated	Male	Female	Male	Female
**Reduced brain weight**	PND 14+	PND 21+	3 weeks^a^ (Bird, Jaenisch); PND 21^b^ – volume by MRI (Jaenisch)	PND 21 ^b^ – volume by MRI; (Jaenisch)
**Hypoactivity/reduced locomotor activity**	PND 21–28+	6 months+	PND 21^c^ (Jaenisch); PND 24^d^ (R168X); 3–8 Weeks^e^^,^^f^^,^^g^ (Bird)	3–8 weeks^e^ (Bird); PND 29–34^h^ (Jaenisch)
**Reduced motor coordination**	PND 21–28+	9 months	PND 29^d^ (R168X)	PND 30^d^ (R168X)
**Abnormal gait**	PND 21–28+	PND 21–28+	3–8 weeks^e^^,^^i^ (Bird); 4 weeks^j^ (Jaenisch)	3–8 weeks^e^ (Bird)
**Breathing irregularities**	PND 21–28+	18 months	PND 15–25^k^ –response to hypoxia (Bird); 4+ weeks^l^ (Bird)	3 weeks^m^ (Bird)
**Seizures**	PND 21–28 (by observation only)	9 months + (by observation only)	5+ weeks^n^ by observation (T158A); PND 40–70°^,^^p^ – by EEG (Bird)	4+ months^d^ – by observation (R168X); 8–12 months^o^^,^^p^ – by EEG (Bird)
**Tremor**	PND 21–28+	N/A	5 weeks^j^^,^^q^ (Jaenisch)	PND 55^r^ (R168X)
**Hypotonicity/reduced muscular strength**	*PND 21–28+	*9–12 months+	PND 22^c^ (Jaenisch); PND 11–21^g^ *increased strength* (Bird)	35–39 weeks^s^ (308); PND 11–21^g^ *increased strength* (Bird)

^a^Belichenko *et al.* 2008;

^b^Ward *et al.* 2008;

^c^Schaevitz *et al.* 2012;

^d^Schaevitz *et al.* 2013;

^e^Guy *et al.* 2001;

^f^Santos *et al.* 2010;

^g^Santos *et al.* 2007;

^h^Nag and Berger-Sweeney, 2007;

^i^Gadalla *et al.* 2014;

^j^Stearns *et al.* 2007;

^k^Voituron *et al.* 2009;

^l^Viemari *et al.* 2005;

^m^Johnson *et al.* 2015;

^n^Goffin *et al.* 2011;

^o^Colic *et al.* 2013;

^p^D’Cruz *et al.* 2010;

^q^Chen *et al.* 2001;

^r^Wegener *et al.* 2014;

^s^Shahbazian *et al.* 2002.

* Preliminary grip strength and wire hang data not presented, addressed in discussion section.

### General RTT phenotype

In females with RTT, disease overtly presents between 6–18 months of age, and symptom progression occurs over a period of years. In XY males, however, mutations in *MECP2* result in fatal neonatal encephalopathy due to the x-linked nature of the disease, or, rarely, a more classical RTT presentation (47–49). Paralleling findings in humans, *Mecp2*^ZFN/y^ rats became symptomatic within weeks of birth and died early, all findings that are corroborated by earlier reports in various mouse models (10–12,20–22,37,50,51).

While male animal models have long provided utility in the study of RTT due to their advanced disease time course as well as a relative lack of phenotypic variability, the additional examination of female animal models is critical for both a comprehensive understanding of disease phenotype, which depends heavily on random X-chromosome inactivation in female patients, and a prediction of therapeutic potential (52,53). Similar to reports in RTT mouse models, we found that *Mecp2*^ZFN/+^ rats became symptomatic on a more variable and lengthened time course than males, with most displaying overt progressive symptoms between 6 and 9 months of age.

### Body weight and brain size in RTT

Females with RTT often display abnormalities in growth, including body weight regulation, leading to the incidence of both overweight and underweight individuals within the disease population (54–56). Additionally, it has long been known that RTT females display microcephaly on a gross anatomic scale, with brain volume generally being reduced by 25% (57–59).

Mouse models of RTT have demonstrated a wide variety of genotype-specific weight changes, with strain background being considered a major determinant of this feature. Similar to two previous reports (14,15), we found that body weight was the same between *Mecp2*^ZFN/y^ rats and WTs at all time points. In contrast, we report a significant increase in *Mecp2*^ZFN/+ ^body weight beginning at 2–3 months of age and continuing through development. Several previous mouse studies also report weight gain in heterozygous females (11,13,42,44).

We additionally found that *Mecp2*^ZFN/y^ wet brain weight was decreased by PND 14, preceding significant findings on other behavioural tests evaluated herein as well as previous reports of reduced brain volume and weight in PND21+ male mice (36,40). In affected female mice, small (4%) reductions in brain volume have been reported as early as PND 21 by magnetic resonance imaging (40), matching our report of sustained reduction in *Mecp2*^ZFN/+ ^brain weight beginning early in development. All of our findings coincide with early abnormalities in ultrasonic vocalization, postural reflex, and breathing patterns reported in several mouse studies (39,60–63).

### Motor dysfunction

Impairments in motor skills are prominent features in RTT, with the majority of patients either never gaining the ability to walk or losing this skill by adulthood (64–66). Subtle alterations in general movements may be observable in infants within the first several months of life (67,68), and various abnormalities in muscle tone are often present both early and late in the disease course (64,65,69).

We observed a decrease in overall locomotor activity as assessed by the open field test in *Mecp2*^ZFN/y^ rats relative to WTs as early as PND 21. *Mecp2*^ZFN/+ ^rats displayed a similar trend, with significance being reached periodically beginning at 6 months of age. This generalized lethargy and reduction in free ambulation has also been observed in male and female RTT mice relatively early in development (10,22,39,70–74).

When testing paradigms that incorporated motivational factors, such as fear of falling or an appealing dark box, were utilized such that motor coordination per se could be more specifically assessed, we observed that *Mecp2*^ZFN/y^ and *Mecp2*^ZFN/+^ rats performed more poorly overall than their WT counterparts. Similar to findings in previous studies (22), *Mecp2*^ZFN/y^ rats demonstrated decreased latency to fall from a rotating rod as early as PND 21. *Mecp2*^ZFN/+ ^females demonstrated reduced latency to fall at 9 months of age; however, this difference did emerge after *Mecp2*^ZFN/+ ^rats became heavier than WTs, making it difficult to definitively determine a weight-independent effect. Previous studies have reported reductions in latency to fall in heterozygous females as early as PND 30 (22), while others have indicated that motor coordination deficits emerge between 5 weeks and 12 months of age (11,13,22,37,39,42,44,73,74).

Gait assessment revealed alterations in the manner in which *Mecp2*^ZFN/y^ and *Mecp2*^ZFN/+ ^rats crossed a glass walkway; namely, they crossed more slowly, took a greater number of steps, and utilized different types of body support mechanisms to accomplish this goal. Trends toward altered features in *Mecp2*^ZFN/+ ^females generally reached significance at only some time points, which was not altogether surprising given the wide phenotypic variability commonly seen in female mouse models of RTT as well as female patients. Previous reports have demonstrated findings of decreased stride length and increased step frequency in male RTT mice as early as 3–4 weeks of age (37,51,71), and gait ataxia as early as 1–4 months of age in heterozygous female mice (10,11). Interestingly, while the hindpaw width of stance was increased in *Mecp2*^ZFN/+ ^rats, a finding that has been previously reported in mice (51,71), forepaw width of stance and paw print areas were not correspondingly increased as may have been predicted from significant average body weight increases in *Mecp2*^ZFN/+ ^rats relative to WTs. The persistence of gait regularity (observed also in (71)) in the presence of these support type variations in both *Mecp2*^ZFN/y^ and *Mecp2*^ZFN/+^ rats seems to indicate that profoundly symptomatic animals are capable of compensating to accomplish motivated ambulation.

### Other physical and behavioural manifestations of disease

#### Malocclusion

The majority of *Mecp2*^ZFN/y^ rats (∼80%), but only 5 *Mecp2*^ZFN/+^ females, demonstrated severe uneven tooth wear necessitating euthanasia during our study. Dental malocclusion has been observed in other RTT models (10,12). We assume that the low incidence of this feature in female rats was due to their possession of one functional *Mecp2* copy; however, malocclusion was previously reported as a “common” occurrence in female mice (12). Rodents’ teeth grow continuously and require constant gnawing to maintain normal wear. Malocclusion may have indirectly resulted from a change in the animals’ ability or desire to feed due to global MeCP2 absence, a feature that might parallel the gastrointestinal autonomic dysfunction and feeding difficulties observed in girls and boys with RTT (48,75,76). Alternatively, malocclusion may directly result from MeCP2 absence in the peripheral tissues related to tooth development and growth; however, given that decelerated weight gain seemed to precede tooth lengthening and the time course of this feature’s development varied significantly, it seems as though the former may be the more probable explanation. Regardless, the development of malocclusion will be critical to consider in any future study utilizing affected male rats. Stringent evaluation of weight gain/loss will be necessary to track potential nutritional deficits, and stratification of affected males into groups based on the development of malocclusion may yield interesting phenotypic findings.

#### Hind limb clasping

The development of hand stereotypies is a key diagnostic feature of classic RTT (5–8,77). The presence of hindlimb clasping upon tail suspension in animal models of RTT has been suggested to recapitulate this principle finding, although this feature is observed in many rodent models of neurologic disease (78–81). In this study, the majority of *Mecp2*^ZFN/y^ and *Mecp2*^ZFN/+^ rats displayed hindlimb clasping at some point during development, as has been previously reported in many mouse models of RTT.

#### Breathing irregularities

RTT patients often develop irregular breathing characterized by periods of hyperventilation interspersed with apnea or breath holds (82). Mouse models of RTT typically report normal respiratory activity under control conditions (inactive, breathing room air) up to PND 20 (63,83) followed shortly thereafter by abnormalities in post-sigh breathing (63), and later hyper- (84,85) or hypo-ventilation (62,83,86,87) with increased tidal volume (86,88) in conjunction with increased apneic frequency and duration (89–91). These respiratory abnormalities are partly recapitulated in juvenile and adult *Mecp2*^ZFN/y^ rats, which demonstrate decreased respiratory frequency, as well as *Mecp2*^ZFN/+^rats, which show increased frequency. However, our evidence suggests that there are species-specific effects of MeCP2 deficiency on respiratory function. While reoccurring apneas are a characteristic breathing feature in RTT patients (82,90,91) and several mouse models of RTT (90,91), our data show that disruption of MeCP2 in rats does not lead to an overtly apneic phenotype. Furthermore, breathing irregularities in RTT mice typically develop after PND 30 and progressively worsen with age (62,63,83). Our data in MeCP2 deficient rats suggest that respiratory problems are not progressive, and indeed in humans, breathing problems may actually be more severe at younger ages (92). Together, these findings suggest that our understanding of respiratory dysfunction associated with RTT is far from complete, and underscore the need for further examination of animal models of this disease.

#### Seizures

EEG abnormalities and seizures are frequently observed in RTT patients, with seizure prevalence being approximately 60% (93–98). In *Mecp2*^ZFN/y^ males, we observed seizures as early as the fourth postnatal week, and most strikingly in symptomatic older *Mecp2*^ZFN/+ ^females upon behavioural testing or handling. Previous RTT mouse studies have reported seizures as early as the 5^th^–7^th^ postnatal week in males (11,13,37,99–101) and 4 months of age in females (22). Although the absence of 24-h video or EEG monitoring made it difficult to define these episodes as seizures and quantify their prevalence, the abnormal behaviour and posture of the rats during these events were clearly reminiscent of seizure activity. The future quantification of seizure activity in this model is warranted, and studies in the rat would be particularly appealing due to the animals’ large size and the advantages that this would confer in electrode implantation and EEG recording.

#### Social aggression

While we did not pursue additional testing measures of social interaction in this model, we did observe many instances of *Mecp2*^ZFN/y^-initiated aggression that resulted in the severe wounding of littermates. These findings may be related to previous reports of altered aggressive or defensive behaviours (17,18,99,102) and anxiety-like behaviours (19,20,22,37,38, 44,50,61) in RTT mouse models as well as in patients (reviewed in (53)). This aggressive behaviour was not observed in *Mecp2*^ZFN/+ ^females.

#### Musculoskeletal features

Abnormal curvature of the spine, while prevalent in RTT patients (103) and reported in previous mouse models (14), was not observed in any rats in our study. Throughout development, however, *Mecp2*^ZFN/y^ and symptomatic *Mecp2*^ZFN/+ ^rats were generally much more easily handled and examined than their WT counterparts due to the display of significant hypotonia. This observation has not previously been described in mouse models of RTT (25), although early hypotonia is well described in human patients with *MECP2* mutations (65,67,104), and several mouse studies have reported alterations in muscular strength in males and females (14,22,37,38,70). A recent study utilizing a conditional MeCP2 knockout model has demonstrated that loss of MeCP2 affects skeletal muscle through a non-cell autonomous mechanism leading to disorganized hypotrophic fibres and fibrosis (105). Preliminary results not presented herein suggest that muscular strength, as assessed by the wire hang and grip strength tests, may be reduced in both juvenile *Mecp2*^ZFN/y^ males and 12 month *Mecp2*^ZFN/+ ^females; however, genotype-specific body weight differences made these results difficult to meaningfully interpret given that the testing paradigms and data analysis rely heavily on body weight. It is clear that muscle tone is significantly altered, whether mediated through a central or peripheral mechanism, in this rat model. The current model offers a clear advantage in that the sheer size of the laboratory rat makes such features easily detectable.

## Concluding Remarks

The characteristics observed in *Mecp2*^ZFN/y^ and *Mecp2*^ZFN/+ ^rats demonstrate many parallels to those displayed by various mouse models as well as in patients with RTT. It is worth noting that the authors of this publication have additional experience working with the Jaenisch null mouse model of RTT (11), and many of the symptoms described herein are more readily apparent and pronounced in the rat than in the smaller laboratory mouse, especially at very early developmental stages. We believe that this study opens the door to a new avenue of research in the RTT field, as it not only characterizes an entirely new species model of this genetic condition, but does so in both male and female MeCP2-deficient rats throughout development. This novel rat model may serve as a critical tool in improving our understanding of disease progression and subsequently aiding in the development of stage-specific therapeutics.

## Materials and Methods

### Subjects

All experimental protocols were in accordance with the NIH guidelines and were carried out with approval from the Animal Care and Use Committee of the University of Alabama at Birmingham (Animal Permit Number: 09409). Every effort was made to minimize pain and discomfort. Animals used in all experiments were littermates bred in our animal facility by crosses of *Mecp2*^ZFN/+ ^females (SD- *Mecp2*^tm1sage^) to wildtype (WT) S100b eGFP (enhanced green fluorescent protein) males obtained from the National Bioresource Project Rat (Japan) (106). This breeding regimen was maintained due to our inherent interest in the study of the role of astrocytes in the pathophysiology of RTT. All rats were genotyped by PCR using DNA obtained from tail snips and Clontech’s Terra PCR Direct Genotyping kit. Primers used were the following: forward 5-GCAGCATCA GAAGGTGTTCA-3 and reverse 5-GACCTCAATGCTGACGGTTT-3. Expected bands were the following: WT = 345bp, *Mecp2*^ZFN/y^ = 274bp, and *Mecp2*^ZFN/+ ^= 345 bp and 274bp (see Fig. 1A). Subjects were sacrificed during the study if they displayed active malocclusion, deteriorating body condition, or if they were required for brain weight evaluation. Time between behavioural evaluations in repeatedly tested animals was always at least two weeks. Studies in male rats were not pursued after active malocclusion presented. Animals were housed under standard conditions of 12-h light-dark cycles and were provided with water and food available ad libitum.

### Western blotting

Brain regions were dissected from whole tissue and sonicated in lysis buffer containing 1M Tris pH 7.5, 10% SDS, and 1:10 protease and phosphatase inhibitors. Equal amounts of protein (10µg and 7µg of protein from male and female animals, respectively) were electrophoresed on 4–20% Mini-PROTEAN TGX precast polyacrylamide gel (BioRad) for 1.5 h at 200V at room temperature and transferred onto methanol-activated PVDF membranes (Millipore) at 100V for 1 h stirring with a cold pack. Membranes were blocked in 10% nonfat milk prepared in TBS-T shaking for 1 h at room temperature, incubated shaking with mouse monoclonal antibodies against the C-terminus of MeCP2 at 1µg/mL (Sigma) in blocking buffer for 2 h at room temperature (female rat cortex) or 4 h at room temperature (male rat cortex and brainstem), washed 3×5 min in TBS-T, and incubated shaking with anti-mouse HRP-conjugated IgG 1:2000 (Santa Cruz Biotechnology, lot #E3113) for 1 h at room temperature. Following 3×5 min washing with TBS-T, blots were developed using Millipore Luminata Classico Western HRP substrate. Clear MeCP2 bands were observed at the expected molecular weight of 75 kD; however, non-specific bands were observed at ∼200, 50 and 40KD, and were present in WT, *Mecp2*^ZFN/y^ and *Mecp2*^ZFN/+^ lanes. For clarity, only the immunoreactive bands at the expected molecular weight for MeCP2 are shown. Blots were then washed 30 min in TBS-T and incubated shaking with chicken polyclonal antibodies against GAPDH 1:2000 (Millipore) in blocking buffer for 35 min, washed 3×5 min in TBS-T, incubated shaking with anti-chicken HRP-conjugated IgY 1:2000 (Santa Cruz Biotechnology, lot #I2612) for 35 min and developed as above. MeCP2 bands were quantified using Licor ImageStudio Version 4.0, normalized to GAPDH, and averaged based on genotype.

### Brain and body growth curves

Animals were weighed on a weekly basis to the 1/10^th^ gram to evaluate overall growth and monitor for any weight loss that might necessitate sacrifice. For ages at which brain weights were evaluated, animals were anaesthetized with CO_2_ and decapitated. Brains were removed from the skull and weighed to the nearest 1/10^th^ gram. For body weight evaluation in males, only those rats that did not go on to develop malocclusion by PND 50 were included in analysis, as decelerated weight gain seemed to precede severe tooth lengthening, and active malocclusion led to weight loss. Brain and body weights for each time point evaluated were averaged based on genotype.

### Generation of symptom scores

Symptom scores (0–9) were generated on a weekly basis during an observation time of approximately 3–6 min per cage based on the summation of the scores from eight characteristics, with a total score of 9 representing a maximally symptomatic animal. General activity level was scored from 0 to 2, with normal activity (0) including cage exploration, interaction with cagemates, and rearing on the hindquarters during experimenter removal of cage lid, and abnormal activity ranging from lethargic cage exploration and no hindlimb rearing (1) to extreme lethargy or a complete lack of movement during the observation period (2). The presence of hindlimb clasping, breathing abnormalities (gasping, “hiccuping” or breath holding), waddling or low-clearance gait, unkempt fur, fur loss, urine staining around the genitals, and porphyrin around the eyes or nose all individually yielded a score of 1. Symptom scores were averaged based on genotype and reported for each age.

### Open field test

One session consisted of placing each rat in the centre of an open field arena (70 × 70cm) surrounded by clear plastic walls and recording for a 4-min period. All movement was captured using a camera mounted above the arena and was quantified by Ethovision XT version 11 software (Noldus). Single-session genotypic values were averaged at each time point reported.

### Rotor rod test

During one testing session, rats were placed on an accelerating rod of a 5.75” diameter (San Diego Instruments) for three training trials with 2 min rest between each, followed by three recorded test trials with 2 min rest between each. Each trial lasted for a maximum of 10 min, with the rod accelerating linearly from 0 to 5 rpm for the first 30 s, 5 to 10 rpm, 10 to 15 rpm, and 15 to 20 rpm for the remaining three 1.5 min segments, and staying at 20 rpm for a maximum of 5 min if any animal remained on the rod. The amount of time for each rat to fall from the rotating rod onto a foam pad 48” below was recorded for each test trial. Latency to fall values from the three test trials were averaged for each animal and within-genotype session averages were calculated at each age reported.

### Catwalk test

All rats were trained with 3 trials on the Catwalk device (Noldus) one week prior to their first testing session. In short, rats were placed at one end of a glass plate of width 10 cm (adults) or 6 cm (pups) enclosed by two opaque walls and a ceiling, and moved toward the other end, which provided an enclosed dark box as a motivating factor for advancement along the surface. During the testing session, trials were video recorded by a camera placed 48.5 cm below the plexiglass surface, thereby capturing the ventral surface of the animals and allowing for measurement of paw placement. For each animal, the first three trials meeting a requirement of <100% within-trial speed variation were kept for analysis. Noldus Catwalk XT version 10.5 software was used to evaluate all gait parameters reported in this study. The Noldus formula used to calculate the regularity index for each trial is as follows: [(NSSP × 4)/PP] × 100%, where NSSP represents the number of normal step sequence patterns and PP the total number of paw placements within the trial. The support type values reported reflect the percentage of time in each trial during which the animal had three paws simultaneously placed on the plexiglass plate. For each animal, the value of each reported parameter was determined as an average of the three recorded session test trials, and these values were averaged based on genotype at each age shown.

### Whole-body unrestrained plethysmography

Respiratory activity was measured using a constant flow whole-body plethysmograph system (Buxco), utilizing animal chambers (PLY3211 and PLY3213) maintained at room temperature and ventilated with air (1000 mL/min). Respiratory-induced changes in chamber pressure were detected using a pressure transducer calibrated before each experiment by the injection of 1mL or 5mL of air (for PLY3211 and PLY3213, respectively) through a Luer-lock syringe according to Buxco manual guidelines. During a testing session (one per animal per age reported), animals were individually placed into a chamber and allowed 30 min to acclimate prior to data recording. Respiratory activity was then recorded for a period of 25 min, and all experiments were performed between 11am and 6pm to minimize potential circadian effects. Tidal volume (TV) (measured in mL, normalized to body weight and corrected to account for chamber and animal temperature, humidity, and atmospheric pressure), minute ventilation (MV) (mL/min/kg), inspiratory time (Ti) and expiratory time (Te) were recorded continuously using the BioSystem XA for Windows software with a logging rate of 5 s. A 1-min period of relative quiescence was selected from continuous data traces for off-line analysis of all respiratory parameters except apneas. Respiratory frequency (breaths per minute; BPM) was analyzed separately using template search event detection in Axon Clampfit 10.2 (Molecular Devices). We confirmed that the 1-min section of data selected for analysis was devoid of any behaviour artefacts, representative of eupneic breathing over the 25 min period, and appeared stable (i.e., limited inter-breath variability) as determined by plotting each breath (n + 1) against the previous breath (n) and constructing a Poincare plot to confirm close clustering of data points in a manner similar to (107).

To estimate respiratory cycle variability we calculated the irregularity score (IS) as previously described (108) IS = 100 × ABS(P_*n*_–P_*n*__−__1_)/P_*n*__−__1_, where IS is the irregularity score for the *n*th cycle, P_*n*_ is its period, P_*n*__−__1_ is the period of the preceding cycle, and ABS is the absolute value. The frequency and duration of apneic events were determined for the entire 25-min recording. Apneic events were conservatively defined as periods with a Te ≥ 3 breath cycles for a juvenile WT (1.4 s) or 3 breathe cycles for an adult male WT (1.6 s).

### Statistics

Data are reported as mean ± standard error of the mean. Statistical analysis was performed using GraphPad InStat 3 software. All parameters in this study were analyzed via within-genotype values at each developmental time point. Between-genotype comparisons were made independently at each age via non-parametric (Mann-Whitney) tests.

## Supplementary Material

Supplementary Data
