# MeCP2 deficiency results in robust Rett-like behavioural and motor deficits in male and female rats

**DOI:** 10.1093/hmg/ddw435

**Published:** 2017-02-13

**Authors:** Kelsey C. Patterson, Virginia E. Hawkins, Kara M. Arps, Daniel K. Mulkey, Michelle L. Olsen

**Affiliations:** 1 Department of Cell, Developmental, and Integrative Biology, University of Alabama at Birmingham, Birmingham, AL, USA; 2 Department of Physiology and Neurobiology, University of Connecticut, Storrs, CT, USA

Post-publication, the authors discovered a scale factor error of 5 affecting volumetric readings from the whole-body plethysmography chamber used for juvenile male rats (PND 21-28) in this study (Buxco PLY3211). As such, reported tidal volume (TV) and minute ventilation (MV) data are erroneously high for this group. This change does not affect the interpretation of this data as the scale factor change is applicable to both groups being compared. The below figure shows the corrected graphical representation of juvenile male rats' TV and MV readings (Fig. 8F and G). All numerical values have been reduced by a factor of 5 to correct for the recognized instrument error. The text of the results section has been updated online to reflect this numerical correction and should be as follows:

***Mecp2*^ZFNΔ/y^ rats display respiratory abnormalities early in development.**

Respiratory activity was measured in juvenile male (PND 21-28), adult male (PND 40+), and aged (18 month) female rats by whole-body plethysmography. We found that under control conditions (awake and breathing room air) juvenile *Mecp2*^ZFNΔ/y^ rats exhibited abnormal breathing compared to WTs (Supplemental Video 10, Fig. 8A and B). Juvenile WTs breathed at a rate of 126.6 ± 5.3 BPM, which is comparable to respiratory rates of similarly aged Sprague-Dawley rats (46); however, age-matched *Mecp2*^ZFNΔ/y^ rats breathed at a rate of only 100.1 ± 4.7 BPM (Fig. 8A and C). The reduction in frequency was due to both an increase in inspiratory time (Ti) (0.20 ± 0.01 s WT vs. 0.25 ± 0.01 s *Mecp2*^ZFNΔ/y^) (Fig. 8D) and expiratory time (Te) (0.28 ± 0.01 s WT vs. 0.37 ± 0.03 s *Mecp2*^ZFNΔ/y^) (Fig. 8E). Despite these changes in respiratory frequency, juvenile *Mecp2*^ZFNΔ/y^ males exhibit an otherwise normal tidal volume (9.1 ± .6 ml/kg WT vs. 10.6 ± .6 ml/kg *Mecp2*^ZFNΔ/y^) (Fig. 8F), minute ventilation (1151.6 ± 85.0 ml/min/kg WT vs. 1073.7 ± 101.1 ml/min/kg *Mecp2*^ZFNΔ/y^) (Fig. 8G), a relatively stable respiratory cycle (irregularity score = 5.0 ± 0.6 WT vs. 5.1 ± 0.6 *Mecp2*^ZFNΔ/y^) (Fig. 8H), and a similar number of spontaneous apneas (Fig. 8I), but of a shorter duration compared to controls (2.5 ± 0.1 s WT; 2.1 ± 0.1 s *Mecp2*^ZFNΔ/y^) (Fig. 8J and B).

**Figure 8.**
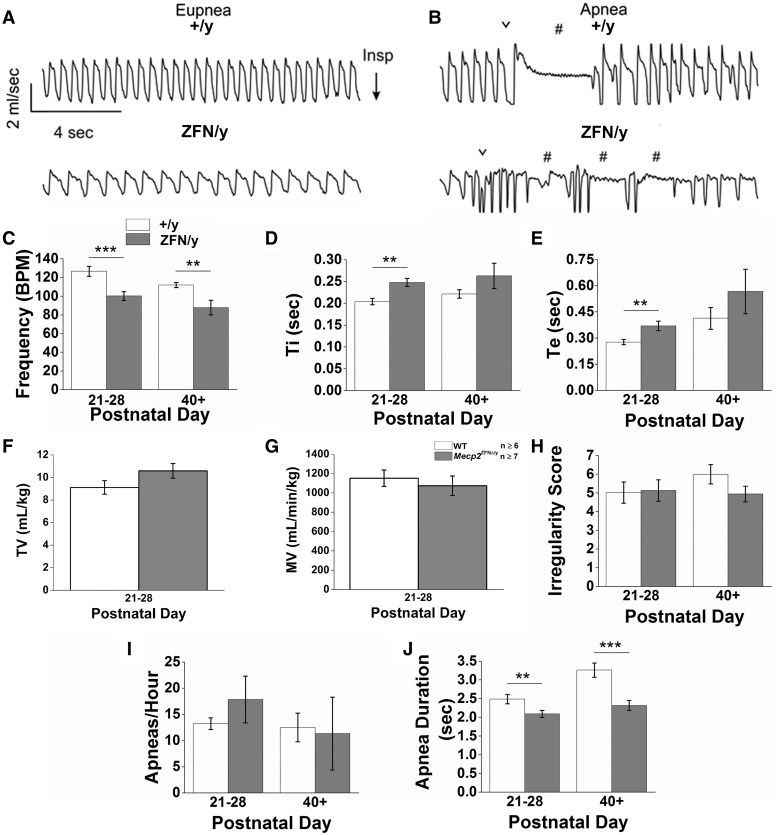
*Mecp2*^ZFN/y^ males display breathing abnormalities consistent with RTT. **(A–B)** Representative raw data plethysmography traces illustrate (A) eupnic breathing and (B) spontaneous apneic events in WT (top tracings) and *Mecp2*^ZFN/y^ (bottom tracings) juvenile males (PND 21–28). Summary data show that juvenile and adult (PND 40+) *Mecp2*^ZFN/y^ rats hypoventilate **(C)** but with no change in TV **(F)**, MV **(G)**, or respiratory irregularity **(H)** (PND 21–28 WT *n = *8, *Mecp2*^ZFN/y^*n = *11; PND 40+ WT *n = *6, *Mecp2*^ZFN/y^*n = *7). Consistent with reduced respiratory frequency, juvenile *Mecp2*^ZFN/y^ rats also showed an increase in Ti **(D)** and Te **(E)**. Data are represented as mean ± SEM, with asterisks representing significant differences (*P < 0.05, **P < 0.01, ***P < 0.001). ∨ designates sigh-like augmented breaths; # identifies apneic events.

