# Supplementary material for: MeCP2 deficiency results in robust Rett-like behavioural and motor deficits in male and female rats
Source: Hum Mol Genet. 2016 Jun 21;25(15):3303–20. doi: 10.1093/hmg/ddw179 (PMC5179928; doi:10.1093/hmg/ddw179)
Supplement: Supplementary Data [file supp_25_15_3303__index.html]

MeCP2 deficiency results in robust Rett-like behavioural and motor deficits in male and female rats — MeCP2 deficiency results in robust Rett-like behavioural and motor deficits in male and female rats — Supplementary Data 

# MeCP2 deficiency results in robust Rett-like behavioural and motor deficits in male and female rats

## Supplementary Data

files

- Supplementary Data - zip file
